# Optimization of *In Vitro* Expansion and Activation of Human Natural Killer Cells against Breast Cancer Cell Line

**Published:** 2020

**Authors:** Farzaneh Peighambarzadeh, Anahita Najafalizadeh, Nafiseh Esmaeil, Abbas Rezaei, Farzaneh Ashrafi, Mazdak Ganjalikhani Hakemi

**Affiliations:** 1. Department of Immunology, Faculty of Medicine, Isfahan University of Medical Sciences, Isfahan, Iran; 2. Hematology Division, Department of Internal Medicine, Isfahan University of Medical Sciences, Isfahan, Iran

**Keywords:** Interleukins (IL), Immunotherapy, Natural killer cells

## Abstract

**Background:**

Regarding to the increase of cancer deaths in recent years and disability of common therapies to eradicate cancers, as well as expansion of Natural Killer (NK) cell therapy, it seems so vital to find new useful therapies against cancers. Breast cancer is the second main cause of cancer death among women. As it is impossible for a majority of patients to receive NK cell therapy, an attempt was made to establish a low-cost and efficient method for expanding and activating NK cells against breast cancer cell line (MCF7).

**Methods:**

NK cells were isolated from Peripheral Blood Mononuclear Cells (PBMCs) applying either MACS based NK cell enrichment kit or antibodies and complement as cytotoxic method. Then, the NK cells were cultured in Stem Cell Growth Medium (SCGM) with feeder layer (irradiated PBMCs) along with PHA or OKT3. IL-2, IL-15 and IL-21 were used to expand NK cells and finally their cytotoxic activity was investigated by flow cytometry.

**Results:**

Highly pure NK cells were obtained and no significant difference between the two isolation methods was found. Using IL-2 plus IL-15, the number of NK cells increased up to100 fold after 16 days. No significant effect was observed after IL-21 treatment.

**Conclusion:**

Our data indicated that cytotoxicity method can be considered a low-cost alternative for NK cell isolation kits. It seems that culturing NK cells for 14 days in either PHA or OKT3 supplemented SCGM medium would be more effective than culturing for 16 days in the presence of IL-21.

## Introduction

As the common therapies of cancers are not able to eradicate the whole cancer stem cells and rather suppress the immune system which leads to cancer relapse, finding a new way to specifically destroy cancer cells without affecting healthy cells and the immune system is a crucial requirement 1,2. Among all types of cancers, breast cancer is the most commonly diagnosed cancer and a major cause of death among women accounting for 15% of cancer deaths among females and 25% of all cancer cases worldwide. Thus, it represents a critical public health problem and there is an urgent need to develop effective treatments against the aggressive subtypes of breast cancer 3–5. MCF7, originally isolated from a 69 year old woman suffering from metastatic breast cancer (Adenocarcinoma), is the most studied cell line to evaluate breast cancer *in vitro*. Its popularity in breast cancer researches reflects its fidelity to many aspects of the cancer in the clinical settings 6.

Based on the immunosurveillance theory, the immune cells aren’t sufficiently powerful in cancerous patients because their anti-tumor activity has been diminished 1,7,8. Thus, restoration of the power of the immune system can be promising 5. In recent years, some immuno therapeutic approaches have been introduced to activate the immune system against cancer cells by strengthening different parts of immune system 9. Adoptive Cell Therapy (ACT) as a type of inactive immunotherapy is an approach to increase, change and enhance the biological activities of immune cells such as Natural Killer (NK) cells 10,11. NK cells as an important part of innate immunity play a key role in destroying the cancer cells. NK cells don’t kill normal host cells because their inhibitory receptors can recognize self MHC class 1 molecules 12.

Nowadays, using NK cells for immunotherapy has provided a new hope for curing cancers because of killing tumor cells without the need to recognize tumor-specific antigens 13. NK cells comprise a small portion of peripheral lymphocytes population and hence, they should be isolated, activated and expanded to a large number, and then to be administered to the cancerous patients 14,15.

In order to isolate NK cells, different studies have used leukapheresis and closed automated systems of cell separation and cultivation and also automated expansion instruments. Several different cytokines, stimulators and mediums such as IL-2, IL-15, IL-12, SCF, IL-7, Phytohaemagglutinin (PHA) and OKT3 in X-VIVO, SCGM, RPMI and GBGM media have been applied for expansion of NK cells so far 16–19. Anti-CD3 (OKT3) and IL-2 can induce secretion of cytokines from T cells which help to activate and expand NK cells and, on the other hand, PHA can impair the immune competence of T cells *in vitro* 20,21. To better expand NK cells, different feeder layers have also been used, such as K562, HWFT, and EBV-LCL cell lines and Peripheral Blood Mononuclear cells (PBMCs) 14.

As the proposed methods are so expensive for majority of patients, especially in developing countries, and the rate of the cancer morbidity and mortality increases in such areas, an attempt was made to establish an efficient and cost benefit, manual method for use in developing countries. The results of this paper may pave the way to develop the possibility of immune cell therapy for low-income patients throughout the world.

## Materials and Methods

### Purification

The PBMCs were isolated using Ficoll Paque (Bio sera, UK) density gradient from whole blood samples of 10 healthy donors after receiving the informed consent. Then, two different methods were applied to purify NK cells:

***Isolation of NK cells with cytotoxicity method:*** 1.5×106 PBMCs along with 7.5 *μg*/*ml* anti CD3 and 15 *μg*/*ml* anti CD19 (CMG, Iran) per 0.5 *ml* RPMI 1640 (Bio-idea, Iran) medium were incubated at 37°*C* for 30 *min*. Then, the rabbit complement (Innotrain, Kornberg, Germany) was added (250 *μl*). Cells were further incubated at 37°*C* for 60 *min* and then, the cells were centrifuged and their purity was confirmed with flow cytometric analysis using anti-human CD56-PE/CY5 (Biolegend, USA) and anti-human CD3-PE (CMG, Iran) 22.

***Isolation of NK cells with MACS:*** Magni SortTM Human NK cell Enrichment kit (Ebioscience, USA) was used for this purpose. A suspension of 1×107 PBMCs in isolation buffer (PBS supplemented with 3% FBS and 10 *mM* EDTA) was made according to the manufacturer’s instruction. Undesired cells such as B, T, and NKT cells were excluded using biotinylated antibody cocktail and streptavidin-coated magnetic beads by negative selection. When undesired cells are bound by antibody and magnetic beads, they stick to the magnetic field and just NK cells remain untouched and can pass the magnetic field. Then, NK cells were eluted and their purity was assessed as explained above.

### NK cell expansion

NK cells purified by MACS were cultivated in two different conditions:

***Expansion of NK cells in SCGM with OKT3:*** Co-culturing of 5×105 NK cells with 5×106 irradiated PBMCs (2500 *rad*), as a simply accessible feeder cell, was done with 10 *ng*/*ml* OKT3 (CMG, Iran), 500 *IU/ml* IL-2 and 10 *ng*/*ml* IL-15 (Ebioscience, USA) in 2ml SCGM (Cell Genix, Germany) medium supplemented with 1% penicillin/streptomycin, 5% pre inactivated AB serum to enhance NK function and 10% FBS (Gibco, USA) in a 25 *cm^2^* culture flask at 37°*C* in 5% CO_2_ in standing position. The whole medium was refreshed (Excluding OKT3) at 2 days intervals and culture was continued for 14 days. Then, their cytotoxic activity and CD107a expression were measured with flow cytometry method. In addition, 100 *ng/ml* IL-21 (Gibco, USA) was added to the cultured cells and cytotoxic activity as well as CD107a expression was assessed again on day 16.

### Expansion of NK cells in SCGM with PHA

Co-culturing of 5×105 NK cells with 5×106 irradiated PBMCs (2500 *rad*) and 1% V/V PHA plus 500 *IU*/*ml* IL-2 and 10 *ng*/*ml* IL-15 was done in 2 *ml* SCGM medium in 25 *cm^2^* culture flask at 37°*C* in 5% CO_2_. Changing the medium was similar to the previous condition. The culture continued for 14 days in standing position. Then, their cytotoxic activity and CD107a expression was measured with flow cytometry method. In addition, 100 *ng*/*ml* IL-21 (Gibco, USA) was added to the cultured cells on day 14 and cytotoxic activity and CD107a expression was assessed again on day 16. Our negative controls in both conditions were NK cells cultured with feeder layer but without any cytokine treatment. Also, NK cells were cultured with the same condition of expansion with OKT3 and PHA but without feeder layer.

### In vitro cytotoxicity assay

On day 0, 14 and 16, the cytotoxic activity of NK cells was assessed against pre-cultured MCF7 cells (Pasteur Institute, Iran). MCF7 cells were grown in RPMI 1640 medium with 10% FBS and 1% pen/strep in culture flasks at 37°*C* and 5% CO_2_ for several days until adhering to the flask and reaching a desired number.

To investigate the NK cells cytotoxic activity, Annexin V/PI apoptosis detection kit (BD bioscience CO, USA) was used. Firstly, the expanded NK cells were co-incubated with MCF7 cells at 10:1 effector: target ratio for 4 *hr* in 24-well plate. Then, according to the manufacturer instructions, the cells were stained with Annexin V (5 *μL*) and PI (5 *μL*) as well as with anti-CD56-PE/CY5.

### CD107a degranulation assay

Lysosomal-associated membrane protein-1 (LAMP-1 or CD107a) surface expression was evaluated by stimulating NK cells with MCF7 cell line. NK cells and target cells were co-incubated with ratio of 10:1 (E:T) and stained with 5 *μl* anti-human CD107a FITC (Ebioscience, USA) for 1 *hr* in 96-well plate. Then, the Monensin solution (Bio Legend, UK) was added to each well and incubated for 4 *hr*. After washing and adding blocker (AB+serum), the cells were washed again and stained with anti-CD56-PE/CY5 and anti-CD3-PE for flow cytometry analysis.

For the negative control, freshly isolated NK cells without any stimulation were co-cultured with MCF7 cells and then CD107a expression and apoptosis were assessed.

### Statistical analysis

Statistical analysis was performed using Kruskal-Wallis test in the case of comparison of purity between two different isolation methods and one way ANOVA was applied in the case of assessing cytotoxic activation and expression of CD107a marker. Statistical significance was set at p≤0.05. All experiments were done three times and their mean and Standard Deviation (SD) values are shown.

## Results

### NK cell isolation

**Figure 1.**
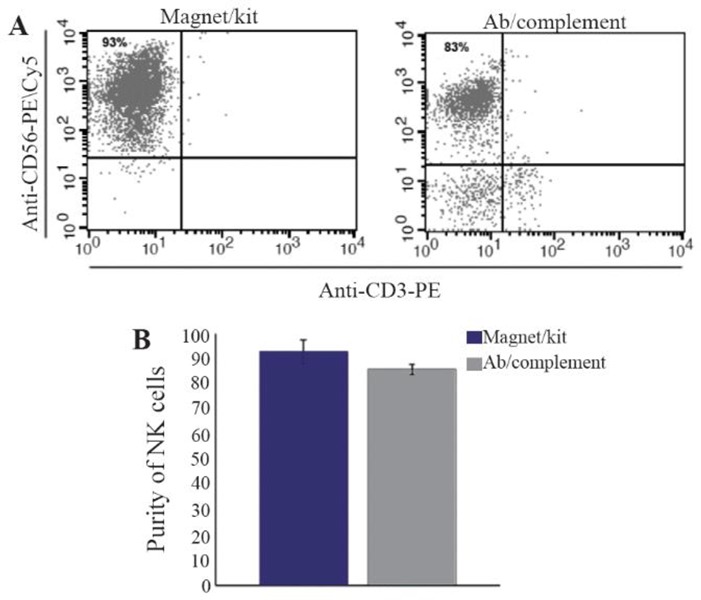
Flow cytometry analysis of two NK cell isolation methods from PBMCs (A). Using MagniSort^™^Human NK cell Enrichment kit, more than 92% purity was detected. We also detected 83% NK cell purity after incubating PBMCs with anti-CD3, anti-CD19 and mouse complement. Bare chart shows no statistically significant difference between the two methods (B).

Using cytotoxicity method, high pure NK cells with low percentage of other mononuclear cells were obtained by this method. Finally, the average purity of NK cells was 83±3.21%. Using Magni SortTM Human NK cell Enrichment kit, NK cells with 92.9±4.71% purity were obtained (Figure 1A). Statistical analysis showed no significant difference between the two isolation methods (p=0.051), (Figure 1B).

### NK cell expansion and activation

**Figure 2.**
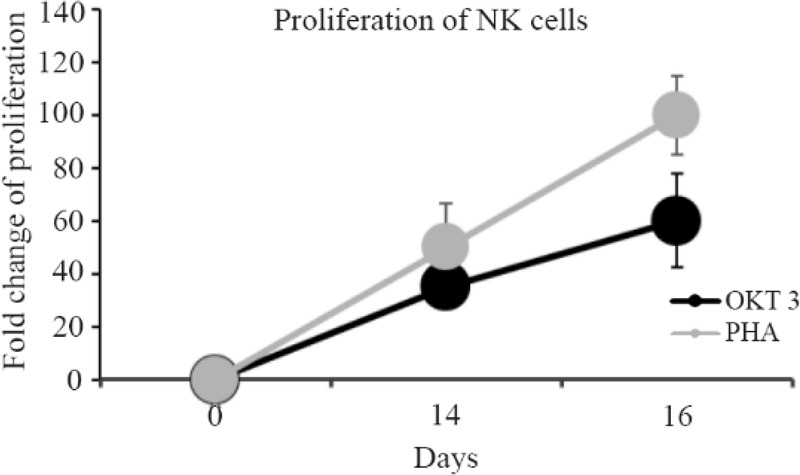
Linear chart shows the comparison of NK cells proliferation stimulated with PHA and OKT3 between days 0, 14 and 16.

1) Stimulation with OKT3: In comparison with day 0, flow cytometry analysis on day 14 and 16 showed 35-fold and 60-fold increase in NK cell numbers, respectively (Figure 2). The differences between day 0 and 14, day 0 and 16 and also day 14 and 16 were statistically significant (p=0.007, p=0.00, p=0.027, respectively). The mean percentage of CD56+CD3-cells was 92.5±2.1% on day 14 and 91.4±3.2% on day 16.

**Figure 3.**
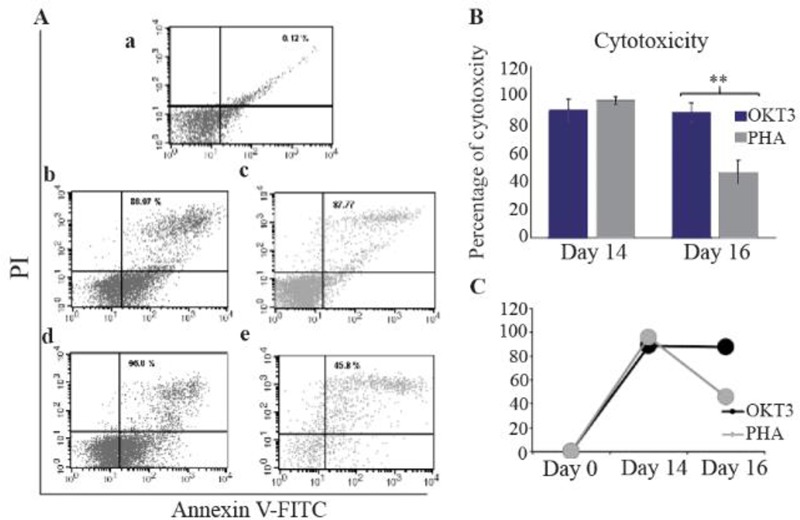
Flow cytometry analysis of NK cells cytotoxicity against breast cancer cell line, MCF7, using Annexin V/PI (A). NK cell cytotoxicity on day 0 (a). NK cell cytotoxicity on day 14 and 16 in the OKT3 treated populations (b and c). NK cell cytotoxicity on day 14 and 16 in the PHA treated populations (d and e). Bare and linear charts (B and C) and show the comparison of NK cells cytotoxicity treated with OKT3 or PHA in different days. Asterisk (*) represents for p<0.05.

On day 0, cytotoxicity of NK cells was 0.12±0.12% while after stimulating with OKT3, cytotoxicity of 88.97±7.97% on day 14 and 87.77±6.55% on day 16 were achieved. NK cell cytotoxicity increased significantly on day 14 and 16 in comparison to day 0 (p= 0.000). There was no significant difference between day 14 and 16 (p=0.83) (Figure 3). The viability of NK cells on day 0, 14 and 16 was 88±0.7, 91±2.7 and 96.16±0.94%, respectively. The difference between day 0 and 14 was not significant (p=0.157) but between day 0 and 16 and between day 14 and 16 was statistically significant (p=0.003, p=0.023, respectively).

The mean apoptosis rate of NK cells was 12±0.8, 5.4±0.5 and 11±0.9% on days 0, 14 and 16, respectively. Compared to day 0, the difference was significant for day 14 (p=0.000). Between day 0 and 16, no significant difference was observed (p=0.306).

**Figure 4.**
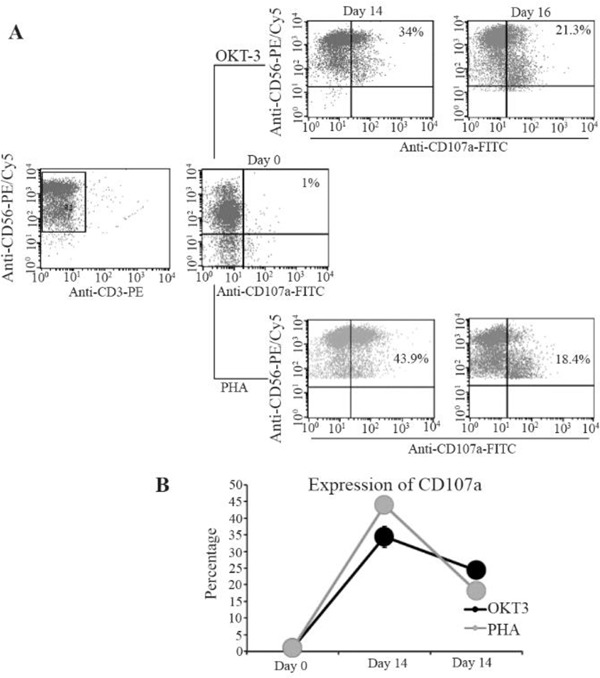
Flow cytometry analysis of CD107a expression on NK cells staining with CD56-PE/CY5 and CD107a-FITC (A) Dot plots show the CD3- CD56+ NK cells were gated. The percent frequency of CD3−CD56+ NK cells that express CD107a marker is indicated in the quadrants in control in compare with PHA and OKT3 treated populations. Linear chart (B) shows the comparison of CD107a expression on NK cells treated with PHA or OKT3 in different days.

After culturing NK cells in SCGM medium, on day 0.85±4.3% NK cells in which 1±0.2% were CD107a positive were detected. After treating NK cells with OKT3, 34±3.2% CD107a positive NK cells remained on day 14 and 24.3±1.7% CD107a positive NK cells on day 16 after treatment with IL-21. So, after treating NK cells with OKT3, CD107a expression was significantly increased on day 14 and 16 in comparison to day 0 (p=0.000, p=0.003) but was significantly decreased on day 16 compared to day 14 (p=0.000), (Figure 4). 2) Stimulation with PHA: on 14th and 16th days, the cells were expanded by an average of 50-fold and 100- fold, respectively (Figure 2). In comparison to day 0, significant increase of expansion was detected on day 14 (p=0.003) and 16 (p=0.000). Also, significant increase was detected between day 14 and 16 (p=0.003). Compared with OKT3 treated NK cells, difference in expansion was not significant on day 14 (p=0.209) while on day 16, the expansion rate was significantly higher (p=0.04).

After treatment with PHA, the cytotoxic activity on day 14 and 16 was 96±2.63% and 45.8±8.5%, respectively. The cytotoxicity of NK cells was significantly higher on day 14 and 16 compared to day 0 (p=0.000 and p=0.001, respectively). NK cell cytotoxicity decreased significantly on day 16 compared to day 14 (p= 0.000), (Figure 3). In comparison with OKT3 supplemented condition, cytotoxicity of PHA stimulated NK cells significantly decreased on day 16 (p=0.002) while no difference was found on day 14 (p=0.22).

The mean viability of NK cells on day 14 and 16 was 95.54±0.43% and 92.28±0.83%, respectively. Significant increase was detected in NK cell viability on day 14 and 16 compared with day 0 (p=0.0001). Viability of PHA stimulated NK cells on day 14 was higher than OKT3 treated ones (p=0.035), while on day 16, viability of OKT3 treated NK cells was significantly more (p=0.006).

Also, 10.7±0.6% apoptotic NK cells were detected on day 14 and 15.5±0.6% on day 16. Compared to day 0 in which 12±0.8% of NK cells were apoptotic, the decrease of apoptosis on day 14 was not significant (p=0.121). However, increase of NK cells apoptosis on day 16 was significantly more than the days 0 and 14 (p=0.002, p=0.000, respectively).

On day 14, from 97±1.7% NK cells, 43.9±2.1% were CD107a positive and on day 16, the percentage of NK cells was 89±3.9% with 18.14±1.86% CD107a positive cells. Thus, CD107a expression increased significantly (p=0.000) on day 14 and 16 in comparison to day 0. The CD107a expression showed significant decrease on day 16 compared to day 14 (p=0.000), (Figure 4). NK cells cultivated as negative control in SCGM medium died on day 7. In comparison with OKT3 treated NK cells, CD107a expression of PHA stimulated NK cells on day 14 was higher (p=0.013), while on day 16, CD107a expression of these cells was significantly lower (p=0.014).

## Discussion

Considering the ability of NK cells in killing cancer cells without pre-stimulation, applying them for cell-based immunotherapies has been increased in recent years 23,24. Several studies for *in vitro* expansion of NK cells have been conducted, so far. However, identifying the optimum NK cell activation and expansion method still continues 25. Hence, an attempt was made to establish a manually cost benefit approach to expand NK cells *in vitro* in order to develop them for low income patients suffering from breast cancer in near future.

In this *in vitro* study, peripheral blood samples were used to isolate the NK cells using two distinct methods of isolation; conventional immunomagnetic method and the cytotoxicity method. Although the purity of NK cells isolated with cytotoxic method was less than the conventional method, statistical analysis showed no significant difference between them (p=0.051). Therefore, it might be possible to apply the cost benefit cytotoxicity method as an alternative with a nearly similar efficiency. However, some modifications are needed to improve the purity. For example, removing monocytes along with T and B cells might be helpful 26. At the moment of this work, because the budget of the project was too limited, it was not possible for us to improve the purity by eliminating monocytes as well as to evaluate these NK cells in all the mentioned experiments. This is now done and the results were promising (data not shown).

As NK cells comprise low percentage of white blood cells population, applying methods to achieve appropriate numbers of these cells is vital 17. Among cytokines, IL-2 has been extensively used, because it is the only cytokine available in clinical grade, and also IL-15 which doesn’t stimulate Tregs 27. Therefore, these two cytokines have been chosen for this work. In the present study, OKT3 or PHA were applied as the stimulators as Imai *et al* detected no sufficient expansion of NK cells while using IL-2 and IL-15 alone 28. Due to irradiation of PBMCs as feeder layer, T cells don’t proliferate but anti-CD3 (OKT3) and IL-2 can induce secretion of cytokines from T cells which can activate and proliferate NK cells. After a while, IL-2-activated NK cells can kill autologous PBMCs 20,21. Therefore, the pure population which remains in the medium, is expanded NK cell and the risk of infusing viable PBMCs is decreased 27.

In one of our experiments, 60-fold proliferation was achieved after co-culturing of irradiated PBMCs and NK cells with OKT3 supplemented SCGM in the culture flasks. After the 16th day, 96% purity was detected which was comparable with Siegler U *et al*’s findings in which, they have achieved 62.7 fold NK cells expansion with near to 100% purity after 19 days 29.

NK cells survival can be decreased after 7 days of culturing with IL-21 30, so IL-21 was added to the medium for 48 *hr* on day 14. After treating OKT3 stimulated NK cells with IL-21, the cytotoxic ability was not increased (87.77%) in comparison with NK cells treated with IL-2 and IL-15 alone (88.97%). Thus, it seems that only IL-2 and IL-15 are sufficient to improve the cytotoxicity of NK cells. Granzin *et al* have shown a beneficial synergism of 500 IU IL-2, irradiated EBV-LCLs as feeder layer and initial administration of IL-21 which resulted in high expansion of NK cells 31. Thus, it seems that IL-21 could be more efficient in expansion of NK cells not in enhancement of their cytotoxicity.

In another experiment, using PHA, instead of OK-T3, IL-2 and IL-15 along with PBMCs as feeder layer, 100-fold expansion of NK cells with negligible percentage of T cells was observed. The expansion of PHA treated NK cells was more than OKT3 treated ones. So, PHA can be a better choice for expansion of NK cells. Imai *et al* have reported just 2 to 5 fold expansion of NK cells after 7 days while T cells comprised 35% of the cell population 28. This might be due to using PHA and IL-2 as the stimuli without any feeder layer in their experiment. It confirms that the role of feeder layer to improve NK cells expansion is indispensable.

On day 14, IL-21 was added to the PHA supplemented SCGM medium to increase the cytotoxicity of NK cells but the cytotoxicity decreased from 96% to 45.8%. Duarte *et al* have found that PHA can impair the immune competence of T cells *in vitro* 32. Secretion of cytokines from T cells present in the PBMCs population is necessary to activate and proliferate NK cells. Thus, co-culturing NK cells and PBMCs along with PHA can lead to increase of activation and cytotoxicity of NK cells because of impaired activity of T cells. This is confirmatory for our findings. However, NK cell proliferation was also increased in our experiment. Using OKT3 or PHA as a stimulator, cytotoxicity increased on day 14 in comparison to day 0. Comparing the treatment with PHA and OKT3, cytotoxicity was equal in both groups on day 14. While the cytotoxic ability of OKT3 treated NK cells was stable on the 16th day, a severe decrease was observed in PHA treated NK cells on day 16. These results show that the enhanced cytotoxic ability of NK cells in the presence of OKT3 is more stable and long lasting comparing stimulation with PHA.

Interestingly, the percentage of viable NK cells cultured with OKT3 (96.16%) was more than those cultured with PHA (92.28%) after 16 days, which means that PHA is probably toxic for NK cells and may kill them after a while. Accordingly, Klö&x00DF; *et al* have detected the 88.5% viable NK cells cultured in SCGM medium after 14 days which is consistent with our results 33. Increased percentage of CD107a expression on day 14 in comparison to day 0 in both PHA and OKT3 stimulated cells was in line with findings of Park *et al* 34. However, the expression of CD107a decreased on day 16 in comparison to day 14. This decrease was much less in OKT3 treated NK cells.

## Conclusion

In this study, it has been shown that isolating relatively high pure NK cells with cytotoxicity method is possible. Irradiated PBMC could be a suitable, cost benefit and accessible option as feeder layer for NK cells expansion. As our data shows, using OKT3, the cytotoxic activity of NK cells, CD107a expression and also NK cell viability increased on day 14 while apoptosis of NK cells decreased accordingly. On day 16, although the viability of OKT3 stimulated NK cells increased in comparison with day 14, the CD107a expression decreased. Other indexes were stable. On the other hand, by applying PHA, an increase of CD107a expression, cytotoxic activity and viability of NK cells on day 14 were detected and NK cell apoptosis decreased. On day 16, the cytotoxic activity and viability as well as CD107a expression of NK cells decreased and accordingly, apoptosis of NK cells increased. Applying IL-21 did not increase cytotoxicity of the NK cells.

## References

[1] Smith AJ Oertle J Prato D. Immunotherapy in cancer treatment. Open J Med Microbiol 2014;4(3):178–191.

[2] Vinay DS Ryan EP Pawelec G Talib WH Stagg J Elkord E Immune evasion in cancer: mechanistic basis and therapeutic strategies. Semin Cancer Biol 2015;35 Suppl:S185–S198. 25818339 10.1016/j.semcancer.2015.03.004

[3] Yu LY Tang J Zhang CM Zeng WJ Yan H Li MP New immunotherapy strategies in breast cancer. Int J Environ Res Public Health 2017;14(1). pii: E68. 10.3390/ijerph14010068 PMC5295319 28085094

[4] Shen J Pan J Du C Si W Yao M Xu L Silencing NKG2D ligand-targeting miRNAs enhances natural killer cell-mediated cytotoxicity in breast cancer. Cell Death Dis 2017;8(4):e2740. 28383557 10.1038/cddis.2017.158 PMC5477582

[5] Shenouda MM Gillgrass A Nham T Hogg R Lee AJ Chew MV Ex vivo expanded natural killer cells from breast cancer patients and healthy donors are highly cytotoxic against breast cancer cell lines and patient-derived tumours. Breast Cancer Res 2017;19(1):76. 28668076 10.1186/s13058-017-0867-9 PMC5493877

[6] Lee AV Oesterreich S Davidson NE. MCF-7 cells--changing the course of breast cancer research and care for 45 years. J Natl Cancer Inst 2015;107(7). pii: djv073. 10.1093/jnci/djv073 25828948

[7] Spanholtz J Preijers F Tordoir M Trilsbeek C Paardekooper J De Witte T Clinical-grade generation of active NK cells from cord blood hematopoietic progenitor cells for immunotherapy using a closed-system culture process. PLoS One 2011;6(6):e20740. 10.1371/journal.pone.0020740 PMC3116834 21698239

[8] Zhou J. Advances and prospects in cancer immunotherapy. New J Sci 2014;2014.

[9] Lim O Jung MY Hwang YK Shin EC. Present and future of allogeneic natural killer cell therapy. Front Immunol 2015;6:286. 26089823 10.3389/fimmu.2015.00286 PMC4453480

[10] Bodduluru LN Kasala ER Madhana RM Sriram CS. Natural killer cells: the journey from puzzles in biology to treatment of cancer. Cancer Lett 2015;357(2):454–467. 25511743 10.1016/j.canlet.2014.12.020

[11] Levy EM Roberti MP Mordoh J. Natural killer cells in human cancer: from biological functions to clinical applications. J Biomed Biotechnol 2011;2011:676198. 10.1155/2011/676198 PMC3085499 21541191

[12] Mandal A Viswanathan C. Natural killer cells: in health and disease. Hematol Oncol Stem Cell Ther 2015;8(2): 47–55. 25571788 10.1016/j.hemonc.2014.11.006

[13] Childs RW Berg M. Bringing natural killer cells to the clinic: ex vivo manipulation. Hematology Am Soc Hematol Educ Program 2013;2013:234–246. 24319186 10.1182/asheducation-2013.1.234 PMC6610030

[14] Selvan SR Dowling JP. “Adherent” versus other isolation strategies for expanding purified, potent, and activated human NK cells for cancer immunotherapy. Biomed Res Int 2015;2015:869547. 26161419 10.1155/2015/869547 PMC4486741

[15] Shook DR Campana D. Natural killer cell engineering for cellular therapy of cancer. Tissue Antigens 2011;78 (6):409–415. 22077621 10.1111/j.1399-0039.2011.01796.x PMC3218564

[16] Granzin M Soltenborn S Müller S Kollet J Berg M Cerwenka A Fully automated expansion and activation of clinical-grade natural killer cells for adoptive immunotherapy. Cytotherapy 2015;17(5):621–632. 25881519 10.1016/j.jcyt.2015.03.611 PMC8725994

[17] Dahlberg CI Sarhan D Chrobok M Duru AD Alici E. Natural killer cell-based therapies targeting cancer: possible strategies to gain and sustain anti-tumor activity. Front Immunol 2015;6:605. 26648934 10.3389/fimmu.2015.00605 PMC4663254

[18] Yang Y Lim O Kim TM Ahn Y-O Choi H Chung H Phase I study of random healthy donor-derived allogeneic natural killer cell therapy in patients with malignant lymphoma or advanced solid tumors. Cancer Immunol Res 2016;4(3):215–224. 26787822 10.1158/2326-6066.CIR-15-0118

[19] Somanchi SS McCulley KJ Somanchi A Chan LL Lee DA. A novel method for assessment of natural killer cell cytotoxicity using image cytometry. PLoS One 2015;10 (10):e0141074. 10.1371/journal.pone.0141074 PMC4619620 26492577

[20] Ahn YO Kim S Kim TM Song EY Park MH Heo DS. Irradiated and activated autologous PBMCs induce expansion of highly cytotoxic human NK cells in vitro. J Immunother 2013;36(7):373–381. 23924789 10.1097/CJI.0b013e3182a3430f

[21] Alici E Sutlu T Björkstrand B Gilljam M Stellan B Nahi H Autologous antitumor activity by NK cells expanded from myeloma patients using GMP-compliant components. Blood 2008;111(6):3155–3162. 18192509 10.1182/blood-2007-09-110312

[22] South AM Grimm PC. Transplant immuno-diagnostics: crossmatch and antigen detection. Pediatr Nephrol 2016; 31(6):897–905. 26139577 10.1007/s00467-015-3145-z PMC5650740

[23] Fujisaki H Kakuda H Shimasaki N Imai C Ma J Lockey T Expansion of highly cytotoxic human natural killer cells for cancer cell therapy. Cancer Res 2009; 69(9):4010–4017. 19383914 10.1158/0008-5472.CAN-08-3712 PMC2716664

[24] Alnabhan R Madrigal A Saudemont A. Differential activation of cord blood and peripheral blood natural killer cells by cytokines. Cytotherapy 2015;17(1):73–85. 25248279 10.1016/j.jcyt.2014.08.003

[25] Domogala A Madrigal JA Saudemont A. Natural killer cell immunotherapy: from bench to bedside. Front Immunol 2015;6:264. 26089820 10.3389/fimmu.2015.00264 PMC4453475

[26] Delirezh N Shojaeefar E Parvin P Asadi B. Comparison the effects of two monocyte isolation methods, plastic adherence and magnetic activated cell sorting methods, on phagocytic activity of generated dendritic cells. Cell J 2013;15(3):218–223. 24027662 PMC3769603

[27] Rezvani K Rouce RH. The application of natural killer cell immunotherapy for the treatment of cancer. Front Immunol 2015;6:578. 26635792 10.3389/fimmu.2015.00578 PMC4648067

[28] Imai C Iwamoto S Campana D. Genetic modification of primary natural killer cells overcomes inhibitory signals and induces specific killing of leukemic cells. Blood 2005;106(1):376–383. 15755898 10.1182/blood-2004-12-4797 PMC1895123

[29] Siegler U Meyer-Monard S Jörger S Stern M Tichelli A Gratwohl A Good manufacturing practice-compliant cell sorting and large-scale expansion of single KIR-positive alloreactive human natural killer cells for multiple infusions to leukemia patients. Cytotherapy 2010;12(6):750–763. 20491532 10.3109/14653241003786155

[30] Skak K Frederiksen KS Lundsgaard D. Interleukin-21 activates human natural killer cells and modulates their surface receptor expression. Immunology 2008;123(4): 575–583. 18005035 10.1111/j.1365-2567.2007.02730.x PMC2433320

[31] Granzin M Stojanovic A Miller M Childs R Huppert V Cerwenka AJO. Highly efficient IL-21 and feeder cell-driven ex vivo expansion of human NK cells with therapeutic activity in a xenograft mouse model of melanoma. Oncoimmunology 2016;5(9):e1219007. 10.1080/2162402X.2016.1219007 PMC5048763 27757317

[32] Duarte RF Chen FE Lowdell MW Potter MN Lamana ML Prentice HG Functional impairment of human T-lymphocytes following PHA-induced expansion and retroviral transduction: implications for gene therapy. Gene Ther 2002;9(20):1359–1368. 12365001 10.1038/sj.gt.3301807

[33] Klöß S Oberschmidt O Morgan M Dahlke J Arseniev L Huppert V Optimization of human NK cell manufacturing: Fully automated separation, improved ex vivo expansion using IL-21 with autologous feeder cells, and generation of anti-CD123-CAR-expressing effector cells. Hum Gene Ther 2017;28(10):897–913. 28810809 10.1089/hum.2017.157

[34] Park KH Park H Kim M Kim Y Han K Oh EJ. Evaluation of NK cell function by flowcytometric measurement and impedance based assay using real-time cell electronic sensing system. Biomed Res Int 2013;2013: 210726. 10.1155/2013/210726 PMC3819884 24236291

